# Minimally invasive versus open intersphincteric resection of low rectal cancer regardless of neoadjuvant chemoradiotherapy: long-term oncologic outcomes

**DOI:** 10.1038/s41598-021-90215-5

**Published:** 2021-05-26

**Authors:** Jung kyong Shin, Hee Cheol Kim, Woo Yong Lee, Seong Hyeon Yun, Yong Beom Cho, Jung Wook Huh, Yoon Ah Park

**Affiliations:** Department of Surgery, Samsung Medical Center, Sungkyunkwan University School of Medicine, 81 Irwon-ro, Gangnam-gu, Seoul, 06351 Korea

**Keywords:** Cancer, Gastroenterology, Oncology

## Abstract

Intersphincteric resection (ISR) is a surgical technique intended to avoid abdominoperineal resection (APR) in patients diagnosed with low-lying rectal cancer. However, the oncologic outcomes of minimally invasive ISR are still controversial. We analyzed the long-term oncologic outcomes of open and minimally invasive ISR. A total of 313 rectal cancer patients who underwent ISR between 2000 and 2014 were analyzed, including 147 in the open surgery group and 166 in the minimally invasive surgery (MIS) group. This study also analyzed 113 patients who received neoadjuvant chemoradiotherapy (nCRT) for advanced lower rectal cancer. Propensity score matching (PSM) was used to correct for differences between the two groups. 5-year disease-free survival (DFS) rate was the primary end point. The length of hospital stay was significantly shorter in the MIS group (9.6 vs. 11.8 days, *p* < 0.001). Differences in overall postoperative morbidity rates between the groups were not significant; however, the rate of surgical site infection was significantly lower in the MIS group (1.2 vs. 10.9%, *p* < 0.001). The 5-year DFS associated with all stages combined in the matched patients were not significantly different: 75.2% in the open group vs. 64.2% in the MIS group (*p* = 0.214). Similar results were found in matched patients treated with nCRT, with 72.0% in the open group and 61.3% in the MIS group (*p* = 0.078) showing DFS. Both minimally-invasive and open ISR for rectal cancer yielded similar 5-year oncologic outcomes. MIS showed statistically significant advantages in some postoperative outcomes such as reduced surgical site infection and shorter hospital stay, and similar long-term outcomes compared with open ISR. This study also suggests that MIS after nCRT for advanced rectal cancer represents a surgical option with similar oncological results.

## Introduction

The contemporary management of rectal cancer is complex, and among several oncological treatment methods available, surgery is one of the most important modalities. Abdominoperineal resection (APR) is one surgical option available for patients with low-lying rectal cancer^1,2^. Total mesorectal excision (TME) was introduced by Heald as the standard surgical approach to rectal cancer, with the aim of improving oncologic outcomes via complete removal of the mesorectum circumferentially. TME is an important concept in rectal cancer surgery^3–5^, however, research into rectal cancer has led to significant advances in its treatment. Nonetheless, sphincter-saving surgery is still considered a challenge which lies primarily in obtaining a negative circumferential resection margin (CRM) with intact TME among patients with very low-lying rectal cancer. Intersphincteric resection (ISR), which preserves sphincter and reduces the incidence of permanent colostomy represents an alternative to APR^6^. ISR is increasingly being accepted for low rectal cancer when combined with neoadjuvant treatment. According to the guidelines of the National Comprehensive Cancer Network, neoadjuvant treatment is generally administered to patients with advanced mid to low rectal cancer^22^. Recently, neoadjuvant treatments have been used for patients with advanced rectal cancer to not only control local recurrence but also preserve the sphincter muscle^3,4^. More recently, the development of minimally invasive surgery (MIS) has increased the laparoscopic surgical options available. A limited number of studies have reported on laparoscopic ISR, however, they involved a small number of patients with limited follow-up duration^7–10^. Few studies have compared the long-term outcomes of laparoscopic and open ISR in low rectal cancer. Due to the paucity of comparative studies investigating long-term oncologic outcomes, the oncological adequacy of laparoscopic ISR has yet to be established.

The purpose of this study was to compare the long-term oncologic outcome of a large cohort undergoing ISR via MIS or by open surgery, for low rectal cancer, irrespective of exposure to neoadjuvant chemoradiotherapy (nCRT).

## Results

### Patient demographics before and after propensity score matching

The clinical characteristics of patients included in this study are listed in Table 1. There were no significant differences in baseline clinical characteristics between the two groups. The average tumor distance from anal verge (mean ± standard deviation) was 3.2 ± 1.5 cm in the MIS group, and 3.0 ± 1.4 cm in the open group (*p* = 0.272).The frequency of nCRT and adjuvant treatment in the open surgery group was higher than in the MIS group. However, there was no difference between the two groups after PSM (Table 1).

**Table 1:** Demographic characteristics of patients treated with and without nCRT.

	Before propensity score matching			After propensity score matching		
	Open (*n* = 147)	MIS (*n* = 166)	*p*	Open (*n* = 118)	MIS (*n* = 118)	*p*
Age (years, median ± (SD)	58 ± 12	57 ± 11	0.770	58 ± 12	58 ± 11	0.879
**Sex, n (%)**			0.552			0.893
Male	89 (60.5)	95 (57.2)		74 (62.7)	73 (61.9)	
Female	58 (39.5)	71 (42.8)		44 (37.3)	45 (38.1)	
BMI (kg/m^2^)	23.4 ± 3.2	23.3 ± 3.5	0.677	23.4 ± 2.8	23.3 ± 3.1	0.700
**ASA score, *n* (%)**			1.000			1.000
< 3	146 (99.3)	165 (99.4)		117 (99.2)	117 (99.2)	
≥ 3	1 (0.7)	1 (0.6)		1 (0.8)	1 (0.8)	
**Underlying, *n* (%)**	68 (46.3)	77 (46.4)	0.982	59 (50.0)	50 (50.0)	1.000
HTN	49 (33.3)	53 (31.9)	0.791	42 (35.6)	41 (34.7)	0.892
DM	20 (13.6)	27 (16.3)	0.511	17 (14.4)	20 (16.9)	0.591
IHD	7 (4.8)	4 (2.4)	0.259	6 (5.1)	4 (3.4)	0.518
COPD	10 (6.8)	4 (2.4)	0.061	9 (7.6)	4 (3.4)	0.154
**Operative period**			< 0.001			< 0.001
2000–2008	58 (39.5)	7 (4.2)		47 (39.8)	5 (4.2)	
2009–2014	89 (60.5)	159 (95.8)		71 (60.2)	113 (95.8)	
Preoperative CEA (ng/mL)	2.8 ± 5.7	2.9 ± 6.7	0.885	3.1 ± 5.9	2.8 ± 6.3	0.697
Preoperative CA19-9 (ng/mL)	13.4 ± 16.2	13.1 ± 16.0	0.881	12.2 ± 7.2	13.7 ± 17.1	0.361
Tumor distance from AV (cm)	3.2 ± 1.5	3.0 ± 1.4	0.272	2.8 ± 1.7	2.9 ± 1.5	0.528
Neoadjuvant CCRT, *n* (%)	71 (48.3)	42 (25.3)	< 0.001	42 (35.6)	42 (35.6)	1.000
Adjuvant treatment, *n* (%)	104 (70.7)	93 (56.0)	0.007	80 (67.8)	78 (66.1)	0.782

*nCRT* neoadjuvant chemoradiotherapy, *MIS* minimally invasive surgery, *BMI* body mass index, *ASA* American society of anesthesiologists, *HTN* hypertension, *DM* diabetes mellitus, *IHD* ischemic heart disease, *COPD* chronic obstructive pulmonary disease, *CEA* carcinoembryonic antigen, *AV* anal verge.

A subgroup analysis of patients was conducted based on nCRT, which was used to treat 71 patients (48.3%) in the open group and 42 (25.3%) in the MIS group (Table 2). Again there were no significant differences in baseline clinical characteristics between the two groups. PSM yielded similar results for variables between the two groups (Table 3).

**Table 2:** Demographics of patients with nCRT.

	Before propensity score matching			After propensity score matching		
	Open (*n* = 71)	MIS (*n* = 42)	*p*	Open (*n* = 42)	MIS (*n* = 42)	*p*
Age (years, median ± (SD)	57 ± 12	55 ± 10	0.341	58 ± 12	55 ± 10	0.158
**Sex, n (%)**			0.846			0.503
Male	42 (59.2)	24 (57.1)		27 (64.3)	24 (57.1)	
Female	29 (40.8)	18 (42.9)		15 (35.7)	18 (42.9)	
BMI (kg/m^2^)	23.1 ± 3.6	23.3 ± 4.7	0.839	22.7 ± 2.6	23.3 ± 4.7	0.123
**ASA score, *n* (%)**			1.000			1.000
< 3	70 (98.6)	42 (100.0)		41 (97.6)	42 (100.0)	
≥ 3	1 (1.4)	0 (0.0)		1 (2.4)	0 (0.0)	
**Underlying, *n* (%)**	33 (46.5)	17 (40.5)	0.562	24 (57.1)	17 (40.5)	0.127
HTN	23 (32.4)	12 (28.6)	0.682	16 (38.1)	12 (28.6)	0.355
DM	7 (9.9)	9 (21.4)	0.101	4 (9.5)	9 (21.4)	0.131
IHD	3 (4.2)	1 (2.4)	1.000	2 (4.8)	1 (2.4)	0.500
COPD	8 (11.3)	0 (0.0)	0.025	7 (16.7)	0 (0.0)	0.012
Preoperative CEA (ng/mL)	2.1 ± 2.7	2.7 ± 2.7	0.268	2.5 ± 3.0	2.7 ± 2.7	0.778
Preoperative CA19-9 (ng/mL)	14.3 ± 21.6	13.3 ± 12.0	0.241	11.6 ± 6.0	13.3 ± 12.0	0.367
Tumor distance from AV (cm)	3.0 ± 1.3	2.9 ± 1.7	0.485	2.6 ± 1.1	2.9 ± 1.7	0.425
Adjuvant treatment, *n* (%)	59 (83.1)	33 (78.6)	0.620	35 (83.3)	33 (78.6)	0.578

*nCRT* neoadjuvant chemoradiotherapy, *MIS* minimally invasive surgery, *BMI* body mass index, *ASA* American society of anesthesiologists, *HTN* hypertension, *DM* diabetes mellitus, *IHD* ischemic heart disease, *COPD* chronic obstructive pulmonary disease, *CEA* carcinoembryonic antigen, *AV* anal verge.

**Table 3:** Pathologic characteristics of patients with nCRT.

	Before propensity score matching			After propensity score matching		
	Open (*n* = 71)	MIS (*n* = 42)	*P*	Open (*n* = 42)	MIS (*n* = 42)	*p*
Size of primary tumor (cm)	2.8 ± 1.4	2.7 ± 1.4	0.712	2.5 ± 1.4	2.7 ± 1.4	0.576
Proximal resection margin (cm)	13.4 ± 5.6	13.3 ± 4.2	0.905	13.9 ± 5.9	13.3 ± 4.2	0.509
Distal resection margin (cm)	1.7 ± 4.4	1.4 ± 2.0	0.659	1.7 ± 5.3	1.4 ± 2.0	0.691
CRM (mm)	4.9 ± 3.8	6.0 ± 4.2	0.335	5.2 ± 2.8	6.0 ± 4.2	0.297
CRM positive, *n* (%)	3 (4.2)	4 (9.5)	0.421	2 (4.8)	4 (9.5)	0.676
**Tumor grade, *n* (%)**			0.202			0.227
Well differentiated	9 (12.7)	11 (26.2)		4 (9.5)	11 (26.2)	
Moderately	54 (76.1)	30 (71.4)		35 (83.3)	30 (71.4)	
Poorly differentiated	3 (4.2)	0 (0.0)		1 (2.4)	0 (0.0)	
Mucinous carcinoma	2 (2.8)	1 (2.4)		1 (2.4)	1 (2.4)	
Signet ring cell	3 (4.2)	0 (0.0)		1 (2.4)	0 (0.0)	
Lymphatic invasion, *n* (%)	12 (16.9)	5 (11.5)	0.591	7 (16.7)	5 (11.5)	0.533
Vascular invasion, *n* (%)	8 (11.3)	3 (7.1)	0.744	2 (4.8)	3 (7.1)	0.500
Perineural invasion, *n* (%)	3 (4.2)	8 (19.0)	0.018	1 (2.4)	8 (19.0)	0.029
**TNM stage, n (%)**			0.317			1.000
0	13 (18.2)	4 (9.5)		4 (9.5)	4 (9.5)	
I	18 (25.4)	17 (40.5)		17 (40.5)	17 (40.5)	
II	21 (29.6)	10 (23.8)		10 (23.8)	10 (23.8)	
III	19 (26.8)	11 (26.2)		11 (26.2)	11 (26.2)	
**Pathologic T stage, *n* (%)**			0.040			0.577
T0	13 (18.3)	4 (9.5)		4 (9.5)	4 (9.5)	
T1	5 (7.0)	3 (7.1)		4 (9.5)	3 (7.1)	
T2	16 (22.5)	18 (42.9)		15 (35.7)	18 (42.9)	
T3	37 (52.1)	15 (35.7)		19 (45.3)	15 (35.7)	
T4	0 (0.0)	2 (4.8)		0 (0.0)	2 (4.8)	
**Pathologic N stage, *n* (%)**			1.000			0.879
N0	52 (73.3)	31 (73.9)		31 (73.8)	31 (73.9)	
N1	14 (19.7)	8 (19.0)		9 (21.4)	8 (19.0)	
N2	5 (7.0)	3 (7.1)		2 (4.8)	3 (7.1)	
Harvested lymph nodes (*n*)	12.0 ± 6.8	10.7 ± 4.9	0.307	11.4 ± 5.8	10.7 ± 4.9	0.576

*nCRT* neoadjuvant chemoradiotherapy, *MIS* minimally invasive surgery, *CRM* circumferential resection margin.

### Pathologic outcomes before and after propensity score matching

As shown in Table 4, the pathological outcomes of the specimens were similar in terms of tumor size, and distal and circumferential margin (CRM). The average tumor size (mean ± standard deviation) was 3.0 ± 1.6 cm in the open group, and 2.7 ± 1.6 cm in the MIS group (*p* = 0.051). The distal resection margins in the open and MIS groups were 3.5 ± 1.6 cm and 2.7 ± 1.6 cm, respectively (*p* = 0.995), and no distal margin was involved in either group. There was no difference in CRM (5.7 ± 3.9 vs. 6.3 ± 3.9, *p* = 0.355), or the proportion of positive CRM values (6.1% vs. 4.8%, *p* = 0.612) between the open and MIS groups. The TNM stages and pathologic T stage were more advanced in the open group, but no difference was found between the two groups after matching (Table 4). Table 3 displays the pathology results of patients treated with nCRT, which were similar to those of the entire group (Table 3).

**Table 4:** Pathologic characteristics of patients treated with and without nCRT.

	Before propensity score matching			After propensity score matching		
	Open (*n* = 147)	MIS (*n* = 166)	*P*	Open (*n* = 118)	MIS (*n* = 118)	*p*
Size of primary tumor (cm)	3.0 ± 1.6	2.7 ± 1.6	0.051	3.0 ± 1.6	2.7 ± 1.5	0.193
Proximal resection margin (cm)	14.1 ± 6.2	13.9 ± 5.3	0.841	14.2 ± 6.5	13.7 ± 4.9	0.557
Distal resection margin (cm)	1.3 ± 1.8	1.4 ± 1.5	0.756	1.6 ± 3.7	1.5 ± 2.4	0.950
CRM (mm)	5.7 ± 3.9	6.3 ± 3.9	0.355	6.1 ± 4.0	6.4 ± 3.7	0.653
CRM positive, *n* (%)	9 (6.1)	8 (4.8)	0.612	8 (6.8)	6 (5.1)	0.582
**Tumor grade, *n* (%)**			0.116			0.120
Well differentiated	23 (15.6)	45 (27.1)		18 (15.3)	33 (28.0)	
Moderately	110 (74.8)	110 (66.3)		91 (77.1)	77 (65.3)	
Poorly differentiated	6 (4.2)	7 (4.2)		4 (3.4)	6 (5.1)	
Mucinous carcinoma	4 (2.7)	2 (1.2)		3 (2.5)	1 (0.8)	
Signet ring cell	4 (2.7)	2 (1.2)		2 (1.7)	1 (0.8)	
Lymphatic invasion, *n* (%)	28 (19.0)	35 (20.6)	0.731	23 (19.5)	28 (23.7)	0.429
Vascular invasion, *n* (%)	15 (10.2)	14 (7.9)	0.473	9 (7.6)	11 (9.3)	0.640
Perineural invasion, *n* (%)	4 (2.7)	17 (9.7)	0.012	2 (1.7)	15 (12.7)	0.002
**TNM stage, n (%)**			0.032			1.000
0	20 (13.6)	16 (9.6)		11 (9.3)	11(9.3)	
I	49 (33.3)	80 (48.2)		48 (40.7)	48 (40.7)	
II	37 (25.2)	26 (15.7)		26 (22.0)	26 (22.0)	
III	41 (27.9)	44 (26.5)		33 (28.0)	33 (28.0)	
**Pathologic T stage, *n* (%)**			0.044			0.869
T0	21 (14.2)	18 (10.8)		12 (10.2)	12 (10.2)	
T1	16 (10.9)	27 (16.3)		15 (12.7)	20 (16.9)	
T2	43 (29.3)	68 (41.0)		42 (35.6)	39 (33.1)	
T3	66 (44.9)	51 (30.7)		48 (40.7)	45 (38.1)	
T4	1 (0.7)	2 (1.2)		1 (0.8)	2 (1.7)	
**Pathologic N stage, *n* (%)**			0.477			0.964
N0	106 (72.1)	122 (73.5)		85 (72.0)	85 (72.0)	
N1	28 (19.0)	35 (21.1)		23 (19.5)	24(20.3)	
N2	13 (8.9)	9 (5.4)		10 (8.5)	9 (7.7)	
Harvested lymph nodes (*n*)	15.3 ± 7.7	13.8 ± 5.6	0.059	13.4 ± 7.6	12.6 ± 4.4	0.313

*nCRT* neoadjuvant chemoradiotherapy, *MIS* minimally invasive surgery, *CRM* circumferential resection margin.

### Perioperative and short-term outcomes before and after propensity score matching

The perioperative and short-term outcomes are shown in Table 5. Looking first at before matching comparisons, although the total operation time was significantly longer in the MIS group than in the open group (237 vs. 194 min, *p* < 0.001), the duration of hospital stay was significantly shorter in the MIS group (9.6 vs. 11.8 days, *p* < 0.001). The rate of diverting loop ileostomy was higher in the open group (90.4 vs. 71.4%, *p* < 0.001). These results were very similar after matching of patients, with the operation time (238 vs. 198 min, *p* < 0.001), the duration of hospital stay (9.5 vs. 11.8 days, *p* < 0.001), and the rate of diverting loop ileostomy (93.2 vs. 71.2%, *p* < 0.001), all highly comparable and significant.

**Table 5:** Perioperative and short-term outcomes of patients treated with and without nCRT.

	Before propensity score matching			After propensity score matching		
	Open (*n* = 147)	MIS (*n* = 166)	*p*	Open (*n* = 118)	MIS (*n* = 118)	*p*
**MIS approach**	–		–	–		–
Laparoscopic surgery	–	130 (78.3)	–	–	92 (78.0)	–
Robotic surgery	–	36 (21.7)	–	–	26 (22.0)	–
Operation time (minutes)	194 ± 67	237 ± 67	< 0.001	198 ± 71	238 ± 66	< 0.001
Stoma formation, n (%)	105 (71.4)	150 (90.4)	< 0.001	84 (71.2)	110 (93.2)	< 0.001
Transfusion, n (%)	9 (6.1)	2 (1.2)	0.018	9 (7.6)	2 (1.7)	0.031
Open conversion, n (%)	–	2 (1.2)	–	–	2 (1.7)	–
Readmission, n (%)	10 (6.8)	16 (9.6)	0.364	6 (5.1)	9 (7.6)	0.423
**Postoperative morbidity**	41 (27.9)	47 (28.3)	0.934	31 (26.3)	32 (27.1)	0.883
Surgical site infection	16 (10.9)	2 (1.2)	< 0.001	12 (10.2)	1 (0.8)	0.002
Postoperative ileus	17 (11.6)	11 (6.6)	0.127	11 (9.3)	6 (5.1)	0.208
Anastomosis site leakage	2 (1.4)	2 (1.2)	1.000	1 (0.8)	1 (0.8)	1.000
Rectovaginal fistula	2 (1.4)	2 (1.2)	1.000	1 (0.8)	0 (0.0)	0.500
Intra-abdominal abscess	2 (1.4)	2 (1.2)	1.000	0 (0.0)	0 (0.0)	1.000
Urinary retention	7 (4.8)	16 (9.6)	0.099	6 (5.1)	14 (11.9)	0.062
Postoperative mortality (< POD30)	0 (0.0)	0 (0.0)	1.000	0 (0.0)	0 (0.0)	1.000
Length of stay (days)	11.8 ± 4.9	9.6 ± 3.5	< 0.001	11.8 ± 4.9	9.5 ± 3.4	< 0.001
Time to diet (days)	2.1 ± 0.8	2.2 ± 0.6	0.433	2.5 ± 1.0	2.1 ± 0.5	< 0.001
Flatus passage (days)	2.5 ± 1.3	1.7 ± 0.9	0.070	2.9 ± 0.7	2.4 ± 1.1	< 0.001

*nCRT* neoadjuvant chemoradiotherapy, *MIS* minimally invasive surgery.

Differences in overall postoperative morbidity rates between the groups were not significant; however, the rate of surgical site infection was significantly lower in the MIS group (1.2 vs. 10.9%, *p* < 0.001). The incidence of anastomotic leakage, rectovaginal fistula, and intra-abdominal abscess were similar between the groups. After PSM, the results were similar and the incidence of surgical site infection was still significantly lower in the MIS group (0.8 vs. 10.2%, *p* = 0.002).

In the nCRT group (before PSM), the mean operating time was longer in the MIS group than in the open group (249 vs. 184 min, *p* < 0.001). A temporary ileostomy was created in 74.6% of patients in the open group and 95.2% of those in the MIS group (*p* = 0.006). The incidence of anastomotic leakage, rectovaginal fistula, and intra-abdominal abscess were similar between groups (Table 6). Similar results were seen after PSM, with longer operation time in the MIS group (249 vs. 194 min, *p* < 0.001) and a higher percentage of ileostomy formation (95.2 vs. 76.2%, *p* = 0.013). Postoperative complications were not significantly different between the two groups and the length of hospital stay was longer in the open group (11.2 vs. 9.0 days, *p* = 0.012).

**Table 6:** Perioperative and short-term outcomes of patients with nCRT.

	Before propensity score matching			After propensity score matching		
	Open (*n* = 71)	MIS (*n* = 42)	*p*	Open (*n* = 42)	MIS (*n* = 42)	*p*
**MIS approach**	–			–		–
Laparoscopic surgery	–	29 (69.0)		–	29 (69.0)	–
Robotic surgery	–	13 (31.0)		–	13 (31.0)	–
Operation time (minutes)	184 ± 51	249 ± 83	< 0.001	194 ± 67	249 ± 83	< 0.001
Stoma formation, n (%)	53 (74.6)	40 (95.2)	0.006	32 (76.2)	40 (95.2)	0.013
Transfusion, n (%)	5 (7.0)	1 (2.4)	0.409	5 (11.9)	1 (2.4)	0.202
Open conversion, n (%)	–	1 (2.4)	–	–	1 (2.4)	–
Readmission, n (%)	5 (7.0)	3 (7.1)	1.000	1 (2.4)	3 (7.1)	0.616
**Postoperative morbidity**	19 (26.8)	8 (19.0)	0.374	9 (21.4)	8 (19.0)	0.786
Surgical site infection	6 (8.5)	1 (2.4)	0.255	2 (4.8)	1 (2.4)	0.557
Postoperative ileus	9 (12.7)	2 (4.8)	0.207	3 (7.1)	2 (4.8)	0.645
Anastomotic site leakage	2 (2.8)	1 (2.4)	1.000	1 (2.4)	1 (2.4)	1.000
Rectovaginal fistula	2 (2.8)	0 (0.0)	0.529	1 (2.4)	0 (0.0)	0.314
Intra-abdominal abscess	0 (0.0)	0 (0.0)	1.000	0 (0.0)	0 (0.0)	1.000
**Urinary retention**	2 (2.8)	3 (7.1)	0.359	1 (2.4)	3 (7.1)	0.616
Postoperative mortality (< POD30)	0 (0.0)	0 (0.0)	1.000	0 (0.0)	0 (0.0)	1.000
Length of stay (days)	11.4 ± 4.6	9.0 ± 3.2	0.005	11.2 ± 4.5	9.0 ± 3.2	0.012
Time to diet (days)	2.0 ± 0.2	2.1 ± 0.6	0.235	2.4 ± 0.7	2.1 ± 0.6	0.099
Flatus passage (days)	2.5 ± 2.1	1.6 ± 0.9	0.651	3.0 ± 0.6	1.6 ± 0.9	< 0.001

*nCRT* neoadjuvant chemoradiotherapy, *MIS* minimally invasive surgery.

### Oncologic outcomes before and after propensity score matching

Median follow-up was 48.8 months in the MIS group, and 62.2 months in the open group (*p* < 0.001). Supplementary Figure S1 shows the survival rates before PSM. Comparative analysis across all patients showed no significant difference between the two groups in 5-year overall survival (OS) (open 89.6 vs. MIS 93.9%, *p* = 0.406) and 5-year disease-free survival (DFS) (open 73.7 vs. MIS 70.0%, *p* = 0.750) rates. When analyzing only groups receiving nCRT, there was no significant difference between the two groups. Five-year OS was 86.3% in the open group and 85.3% in the MIS group (*p* = 0.929) and 5-year DFS was 70.2% in the open group and 61.3% in the MIS group (*p* = 0.065) (Supplementary Fig. S2).

**Figure 1:**
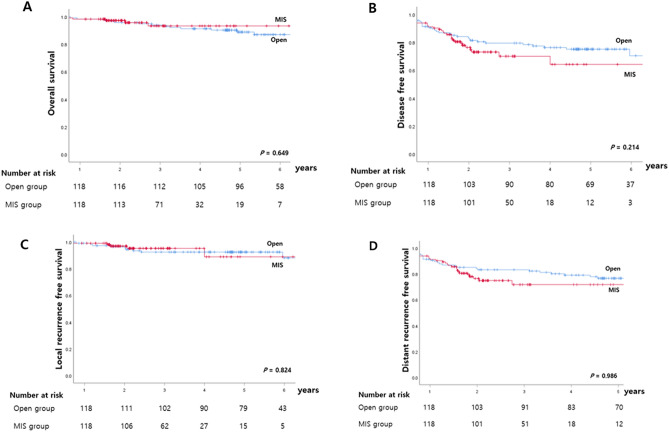
Five-year survival rates according to the operative approach in matched patients. (**A**) Overall survival, (**B**) disease-free survival, (**C**) local recurrence-free survival, (**D)** distant recurrence-free survival.

**Figure 2:**
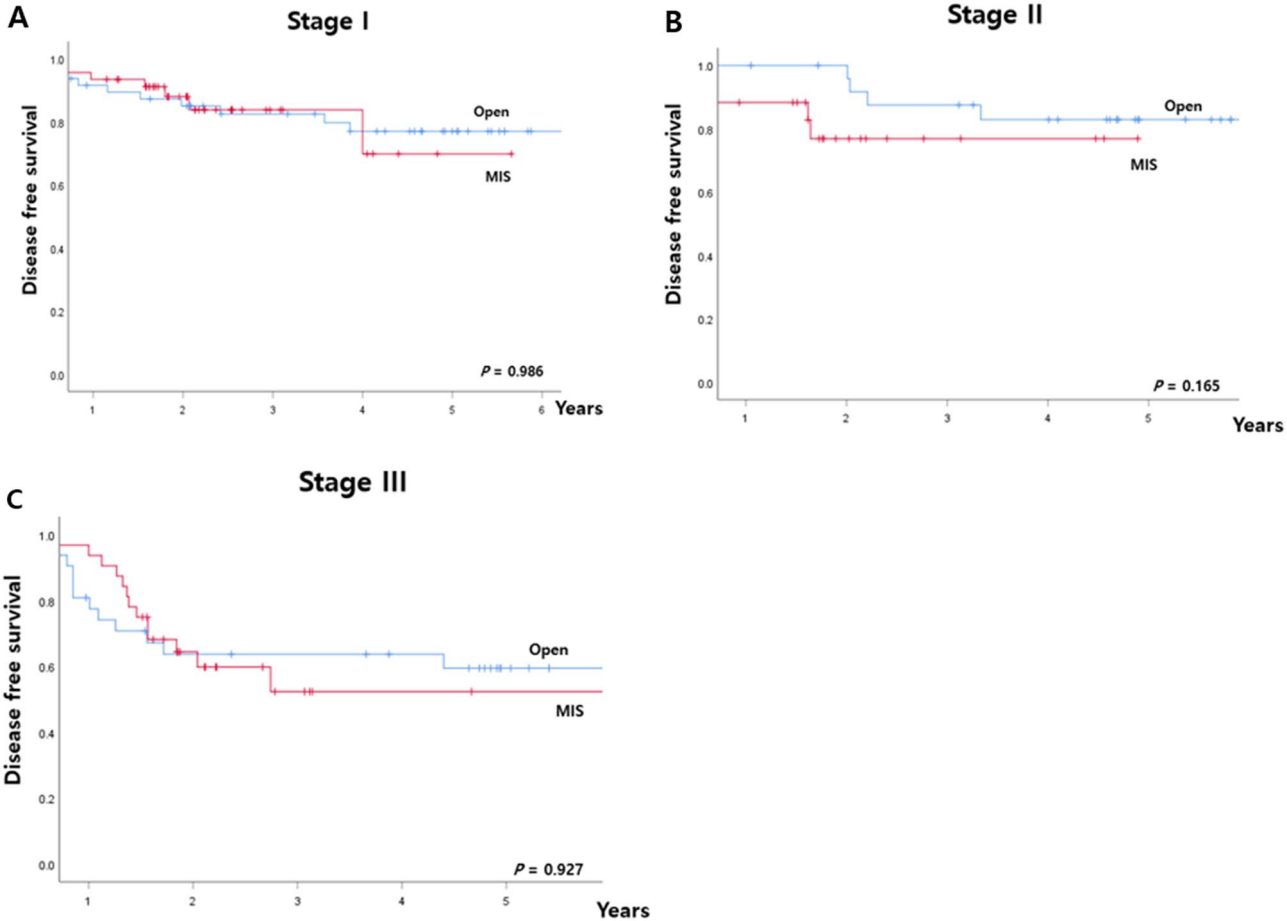
Five-year disease-free survival rates according to operative approach in matched patients (**A**) stage I, (**B**) stage II, (**C**) stage III disease.

After PSM, the 5-year OS rate was 88.0% in the open group and 95.6% in the MIS group (*p* = 0.155). There were no differences in rates of 5-year DFS and local recurrence-free survival (LRFS) (Fig. 1). The 5-year DFS for all stages combined was 73.7% in the open group, and 70.0% in the MIS group (*p* = 0.748). The 5-year DFS rates according to TNM stage in the open and MIS groups showed no significant differences (stage I: 77.5 vs. 82.4%, *p* = 0.223; stage II: 79.4 vs. 82.4%, *p* = 0.368; stage III: 54.1 vs. 48.8%, *p* = 0.948) (Fig. 2). No recurrence was observed at the trocar or mini-laparotomy site. Five patients (3.0%) in the MIS group experienced local recurrence (five pelvic side-wall tumors) compared with nine patients (6.1%) in the open group (six pelvic side-wall tumors, three anastomotic sites).

**Figure 3:**
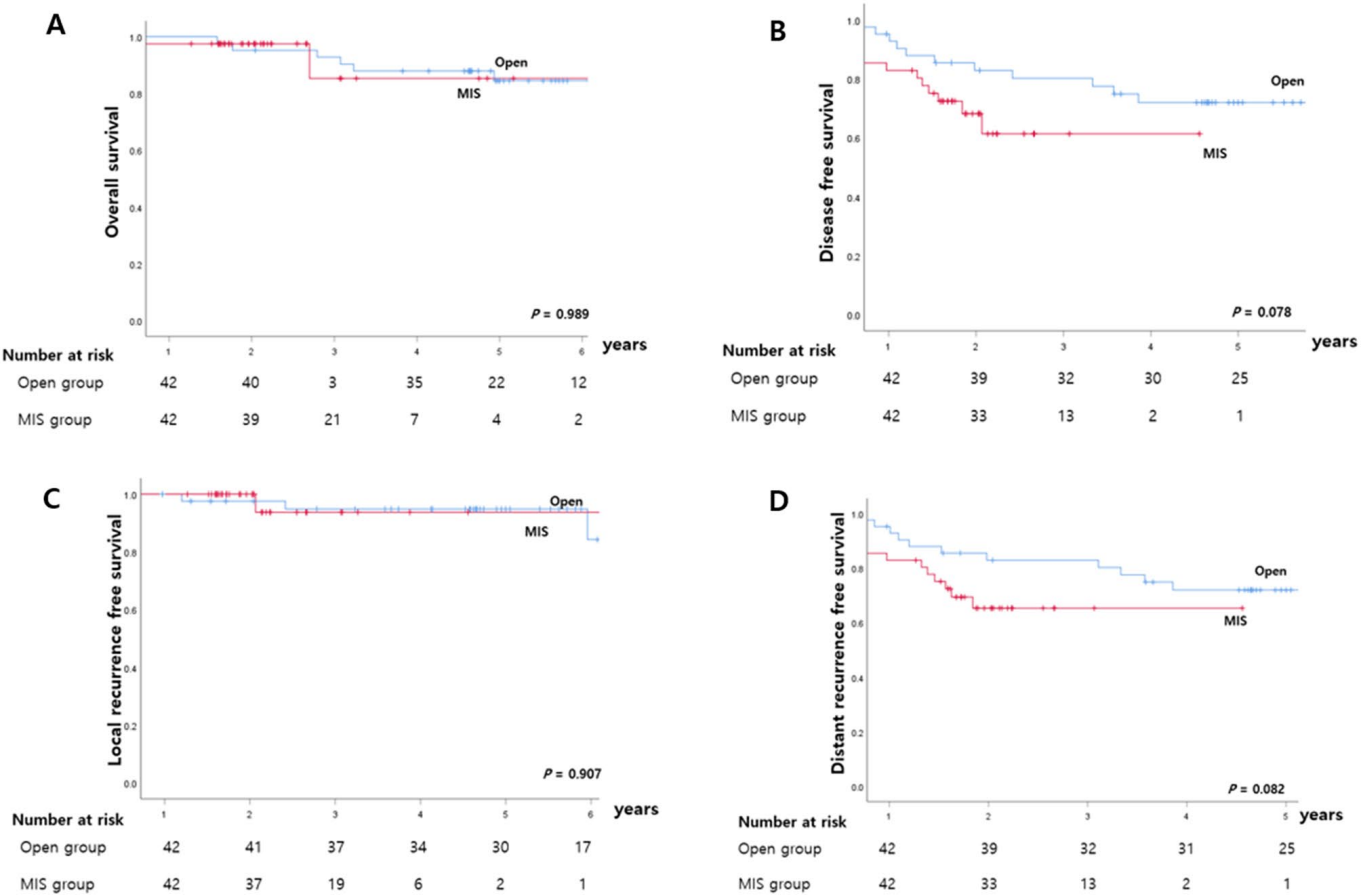
Five-year survival rates of matched patients who underwent nCRT: (**A**) overall survival, (**B**) disease-free survival, (**C**) local recurrence-free survival, (**D**) distant recurrence-free survival.

There were also no differences in the rates of 5-year OS and DFS in the matched nCRT group (Fig. 3). The 5-year OS rate was 86.3% in the open group and 85.3% in the MIS group (*p* = 0.929). The 5-year DFS rate also showed comparable results, with 70.2% in the open group, and 61.3% in the MIS group (*p* = 0.069). Local recurrences occurred in one patient (2.3%) belonging to the MIS group and six patients (8.4%) included in the open group.

## Discussion

Currently there are several studies that have reported on short-term benefits of laparoscopic surgery for rectal cancer^11–13^. However, these studies involved relatively fewer patients who received nCRT for advanced low rectal cancer. Laparoscopic surgery is considered technically challenging in patients with very low rectal cancer because of the difficulties associated with pelvic dissection; with obtaining a negative CRM upon intact total mesorectal excision, and with sphincter preservation.

Remarkably few studies have demonstrated the role of sphincter-saving laparoscopic surgery in patients with low rectal cancer and these have been based on limited follow-up results^7,10^. To our knowledge, this is the one of the largest studies conducted and includes more than 300 curative cases of ISR for rectal cancer. The study compares long-term oncologic outcomes of patients undergoing MIS and open surgery. We found similar postoperative morbidity and mortality with no significant differences in 5-year DFS and LRFS rates between the two groups. As well, the results showed that MIS with nCRT was effective in treating locally advanced lower rectal cancer.

ISR entails resection of the internal sphincter either completely or partially, and restoration of bowel continuity, with good surgical, oncological, and functional outcomes. Compared with the open approach, laparoscopic ISR for rectal cancer, although technically demanding, provides optimal pelvic dissection while maintaining an intact fascia plane, and enhancing the view within the deep pelvic cavity. Laparoscopic ISR yields similar oncologic outcomes, reducing postoperative pain and shortening recovery time^7,8,10^.

One of the concerns regarding MIS is the technical difficulty associated with the surgical procedures. Previous studies reported that the rates of conversion to open surgery were 3.0–21.8% and conversion was associated with a poor prognosis^7,8,10–12^. In this study, the conversion rate was only 1.2% and this was fundamentally attributed to the surgeon’s cumulative experience and skills with laparoscopic technique.

Positive CRM is defined by tumor cell involvement occurring within 1 mm of the circumferential border, and is one of the predictors of recurrence and survival in patients with rectal cancer^13^. The rate of positive CRM in both groups was similar or lower than the previously reported rate of 3–15.5%^8,14–16^. In this study, the CRM was longer in the MIS than in the open group (6.3 ± 3.9 vs. 5.7 ± 3.9, *p* = 0.355), and the rate of margin positivity was lower (4.8% vs. 6.1%, *p* = 0.612), although these differences were not statistically significant. A potential explanation may be that the precise operative field view during MIS facilitates complete mesorectal excision with maintenance of adequate circumferential margin. The distal resection margin was slightly shorter in the MIS group than in the open group. However, many previous studies have challenged the 2-cm distal margin rule, and currently a distal margin of 1 cm is considered adequate for optimal oncologic outcomes^6,17^.

In a majority of previous studies comparing open and laparoscopic ISR, 3-year DFS was used as the primary outcome^7,9,18^. Studies investigating open ISR reported that the local recurrence rate ranged from 0 to 10.6% and the DFS rate was 66.7–77.0%^7,8,14,19,20^. Few studies compared oncologic outcomes between laparoscopic and open groups. Park et al. comparing laparoscopic and open ISR groups demonstrated similar 3-year local recurrence (2.6 vs. 7.7%, *p* = 0.184) and DFS rates (82.1 vs. 77.0%, *p* = 0.523)^7^. Laurent et al. reported no difference in 3-year local recurrence (5 vs. 2%, *p* = 0.349) and 5-year DFS rates (70 vs. 71%, *p* = 0.349) between laparoscopic and open ISR groups^8^. In this study, the 5-year DFS (70.0 vs. 73.7%, *p* = 0.748) and 5-year local recurrence rates (9.3 vs. 9.9%, *p* = 0.502) were similar in the two groups. As well, similar rates of local recurrence and DFS were found when stratified by the TNM stage.

Many surgeons have concerns about the safety of laparoscopic ISR for the treatment of locally advanced rectal cancer such as TNM stage III. nCRT is generally administered to patients with advanced mid-lower rectal cancer and unfortunately the edematous tissue and fibrotic changes induced by nCRT interfere with pelvic dissection^21^. However, our data shows that MIS ISR was comparable to open ISR in patients with locally advanced low-rectal cancer in terms of short- and long-term outcomes.

The limitations of this study are the retrospective format and lack of analysis of functional outcomes. The PSM was used to reduce the selection bias, but because of the retrospective study design, bias may still persist. Another limitation is differences in the timing of operations and the duration of follow-up. Because there were a lot of open surgeries in the early period of the study, the duration was longer in the open group because MIS was performed mostly later. This difference in the follow-up period may have affected the oncological results. Despite these limitations, this study analyzed the long-term oncologic outcomes of a large number of patients who underwent open and MIS ISR at a large-volume colorectal cancer center.

In conclusion, both minimally-invasive and open ISR for rectal cancer yield similar short- and long-term outcomes. MIS for low rectal cancer showed several short-term advantages and similar long-term outcomes compared with open ISR.

## Patients and methods

All consecutive patients undergoing R0 resection for low rectal cancer between January 2000 and December 2014 were analyzed. This study was approved by the Institutional Review Board (IRB) of Samsung Medical Center (IRB No. 2020-02-164). Written informed consent was waived by the IRB due to its retrospective nature. During the study period, 313 patients who underwent ISR were enrolled. Patients were excluded if they had recurrent or metastatic cancer, previous chemotherapy or pelvic radiotherapy, or hereditary rectal cancer. Contraindications included tumor invasion of the external sphincter or levator ani muscle. MIS techniques included conventional laparoscopic surgery, single-incision laparoscopic surgery, and robotic surgery. Of these, 166 surgeries were performed via MIS and 147 operations via open procedures.

This study was conducted in accordance with nCRT guidelines. Among the 313 patients, 113 underwent nCRT including 71 via open and 42 via MIS approaches.

Initial work-up including abdominopelvic computed tomography (APCT) scan, chest CT scan and rectum magnetic resonance imaging. Patients with a locally advanced T3 or T4 stage, and nodal involvement underwent nCRT. Radiotherapy was performed using a dose of 50.4 Gy in 28 fractions for six weeks. Chemotherapy regimens were conducted based on 5-FU. The average interval between surgery and nCRT was 6 to 8 weeks. Adjuvant treatment was performed in pathologic node-positive cases and in patients treated with nCRT.

Short-term outcomes were defined as postoperative complications, such as surgical site infection, anastomosis site leakage, and postoperative mortality. Long-term outcomes included 5-year survival rates, including overall survival (OS), disease-free survival (DFS), local recurrence free survival (LRFS), and distant recurrence-free survival (DRFS) rates. OS is the survival rate after curative surgery, whereas DFS is defined as the survival rate without local recurrence or distant metastasis after curative surgery. LRFS indicates the survival rate without local recurrence after curative surgery.

Short-term and long-term oncologic outcomes were compared between the MIS and open groups. The patients were followed every three months via laboratory testing including tumor markers. Chest CT, and abdominopelvic CT scans were performed every six months.

### Statistical analysis

We used SPSS for Windows version 23.0 (SPSS, Chicago, IL, USA) for analysis. Chi-square, Mann–Whitney U, or Fisher’s exact tests were used to analyze differences between the two groups. The Kaplan–Meier method was used for survival rate analysis. Statistical significance was defined by a p value less than 0.05. There were significant differences between the two groups, which affected survival rates. We used PSM to adjust for factors, such as age, sex, neoadjuvant and adjuvant treatment, TNM stage, pathologic T and N stage, and lymphatic/vascular/perineural invasion. We analyzed matched patients to reduce the bias substantially. An event refers to the occurrence of death, recurrence, local recurrence, and distant recurrence in OS, DFS, LRFS, DRFS, respectively. Date refers to the period after which the event occurred. Censor means data that could not be determined during the study period whether an event occurred or not. This study included a patient that did not experience a relevant outcome. This was a patient lost to follow-up during the study period, and one other patient experienced a different event that made further follow-up impossible.

### Surgical techniques

Both open and MIS techniques were performed by six expert colorectal surgeons working in this hospital. More than 1,500 laparoscopic surgeries (conventional and single-port) and more than 100 robotic surgeries are performed for primary colorectal cancer at this institution annually. Standard total mesorectal excisions (TME) of the levator ani plane and the anorectal junction were performed, exercising caution to preserve the bilateral hypogastric nerve and neurovascular bundle. The intersphincteric plane between the puborectalis and the internal sphincter was dissected as caudally as possible under direct vision. Using a transanal approach, circular incisions were made at least 1 cm below the distal margin of the mass. The specimen was extracted through the anus. Bowel anastomosis was performed via hand-sewing colo-anal anastomosis, including straight, colonic J pouch or coloplasty methods.

## Supplementary Information

Supplementary Figure 1.

Supplementary Figure 2.

Supplementary Figure legends.
