# Supplementary figures and images for: Minimally invasive versus open intersphincteric resection of low rectal cancer regardless of neoadjuvant chemoradiotherapy: long-term oncologic outcomes
Source: Sci Rep. 2021 May 26;11:11001. doi: 10.1038/s41598-021-90215-5 (PMC8155052; doi:10.1038/s41598-021-90215-5)

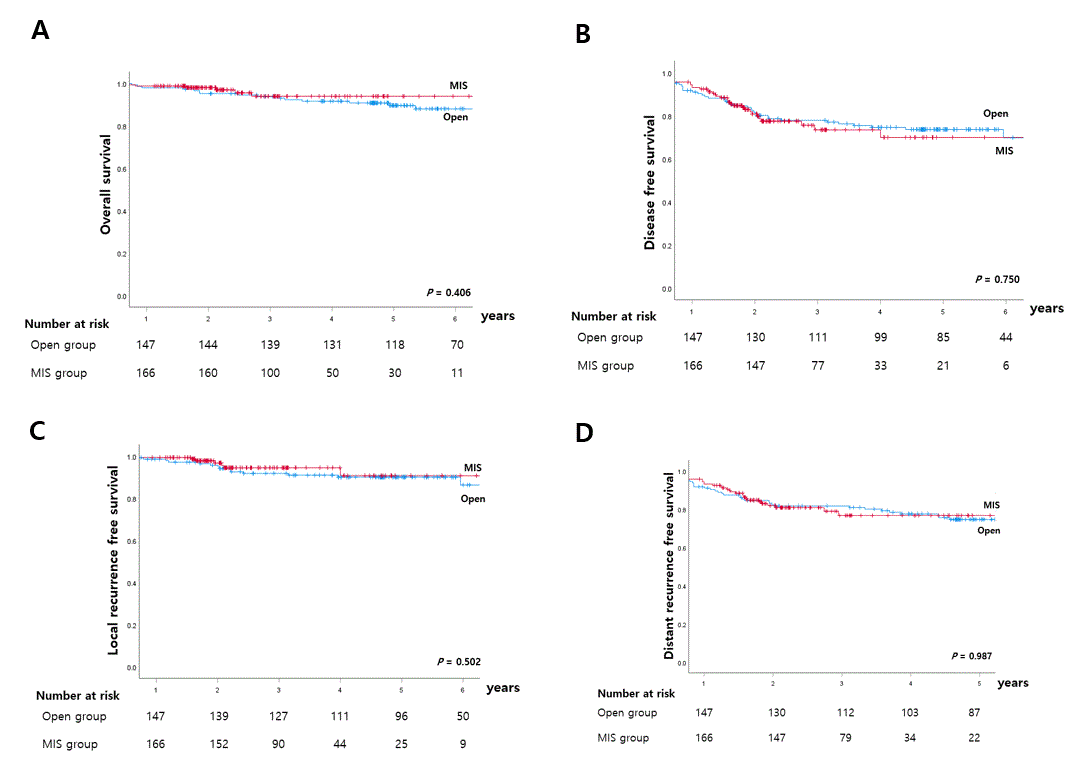

Supplement: Supplementary file 1 — Supplementary Figure 1. [file 41598_2021_90215_MOESM1_ESM.gif]

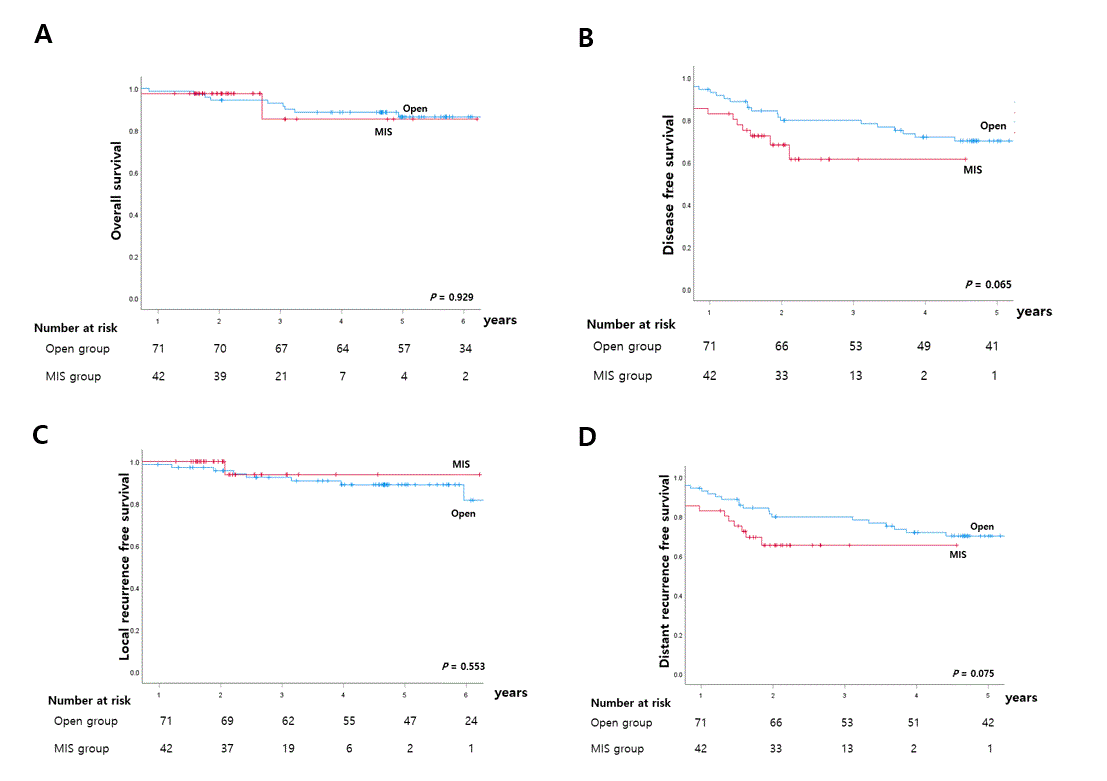

Supplement: Supplementary file 2 — Supplementary Figure 2. [file 41598_2021_90215_MOESM2_ESM.gif]
