# Supplementary material for: Minimally invasive versus open intersphincteric resection of low rectal cancer regardless of neoadjuvant chemoradiotherapy: long-term oncologic outcomes
Source: Sci Rep. 2021 May 26;11:11001. doi: 10.1038/s41598-021-90215-5 (PMC8155052; doi:10.1038/s41598-021-90215-5)
Supplement: Supplementary file 3 — Supplementary Figure legends. [file 41598_2021_90215_MOESM3_ESM.docx]

**Supplementary figure 1.** Five-year survival rates according to the operative approach before PSM. (**a)** overall survival, (**B)** disease-free survival, (**C)** local recurrence-free survival, (**D)** distant recurrence-free survival.

**Supplementary figure 2.** Five-year survival rates of patients with nCRT before PSM. (**a)** overall survival, (**B)** disease-free survival, (**C)** local recurrence-free survival, (**D)** distant recurrence-free survival.
